# An Evolutionary Perspective on the Importance of Community Relations for Quality of Life

**DOI:** 10.1100/tsw.2009.73

**Published:** 2009-07-13

**Authors:** Bjørn Grinde

**Affiliations:** Norwegian Institute of Public Health, Oslo, Norway

**Keywords:** community relations, human behavior, evolution, happiness, quality of life, alternative living, intentional communities

## Abstract

The evolutionary perspective is relevant for the study of quality of life in that the brain, including its capacity for positive and negative states of mind, has been shaped by the forces of evolution. The present text uses this perspective to discuss three questions related to the observation that human interactions are a particular important factor for well-being: (1) What is known about the inherent nature of our social propensities? (2) Is the present situation responsible for a suboptimal quality of life? (3) Are there alternatives to the organization of mainstream Western society? Based on this discussion, the question is raised as to whether it is possible to suggest improvements. Briefly, it seems possible to create conditions that enhance social relations and to the extent that happiness is considered an important objective, this is a relevant endeavor.

### INTRODUCTION – QUALITY OF LIFE IN A BIOLOGICAL PERSPECTIVE

Quality of life is a parameter that typically includes several factors, the more important ones being happiness (measured as subjective well-being by means of questionnaires) and health[1,2]. The present focus will be on happiness; however, the discussion is relevant for health and the broader definition of quality of life as well.

Happiness as a subject of serious research has primarily been developed in the tradition of the social sciences. The central topic is correlates between environmental factors, or ways of living, and well-being. One of the conclusions arising from these studies is that social connections are of paramount importance for both health and happiness, while money, beyond what is required for basic subsidence, carries less weight, even in poorer countries[3,4,5,6,7,8]. The significance of social networks calls for an explanation. Understanding why this factor has such a large impact on happiness is relevant for the purpose of pointing society in the direction of improved quality of life.

The social science approach is required to find correlates between behavioral parameters and mental qualities, such as happiness. In order to explain these correlates, a biological perspective may offer a useful supplement. An understanding of how evolution has shaped the human brain opens for complementary insight into both social behavior and quality of life. The author has previously used the term *Darwinian Happiness* for an evolutionary-based model of what constitutes happiness[9,10]. Briefly, the features evolution has added to the human brain that impact on well-being can be divided into two types of *brain modules*. First, the capacity to experience positive and negative sensations and emotions, also referred to as *brain rewards* and *brain punishment*, is highly relevant[11,12]. This capacity evolved primarily for the purpose of directing the individual either towards or away from particular behavior, for example, respectively, eating and not damaging the body.

Second, the other type of module is more elusive, but concerns what is referred to as a *default state of contentment*. It is in the genes' interest to reside in an individual characterized by a content and optimistic mind, simply because this situation is more likely to cater to survival and procreation. With a positive state of mind, the individual stands a better chance of finding food and a partner. Thus, in the absence of punishing emotions and sensations, evolution has shaped a healthy mind to be in a state of contentment. Lykken[3] offers a more comprehensive discussion on the innate positive frame of mind (see also Table 1).

**TABLE 1**
**Darwinian Happiness***

| Brain Feature | Stress | Social Life |
|---|---|---|
| 1. Encouragements | | |
| a. Rewarding | Reduced pleasure | Possibly the most potent source |
| b. Punishing | Not obvious | Potential for negative feelings |
| 2. Contentment | Highly negative | Good social life important |

*Features of the brain relevant for quality of life. Schematic description of possible impact of stress and social life.

A brief note on brain modules is called for. The concept, as outlined by Nesse[13], is a useful tool when describing a biological theory of happiness because it reflects the way evolution operates. Modules do not necessarily correlate with distinct neurological features. They are defined in functional terms and, as such, are units of selection rather than of anatomy. In other words, modules are utilities shaped by evolution to care for some task or problem required for survival and procreation. The actual physical correlate may be widely dispersed within the brain, shared with other modules, and may involve functions outside the nervous system, such as endocrine glands.

Brain punishments typically involve the activation of modules associated with avoidance behavior. Physical pain is an obvious example, but emotions such as anxiety and low mood also fall into this category and constitute a more challenging problem as to quality of life. These two emotions presumably evolved, at least partly, to help us avoid danger and evade conflicts or misfortune, respectively; i.e., to teach the individual not to end up in situations that trigger negative emotions[14].

The term *discord* covers another important concept. Deviations from the way of life we are genetically designed for, sometimes referred to as the *Environment of Evolutionary Adaptation*, or EEA, have been referred to as *mismatches*[15]. Some mismatches are beneficial, such as sleeping on a mattress instead of on the ground, while others may contribute to disease or reduced quality of life. The word discord is used for those mismatches that have a negative impact; i.e., they cause some form of "stress", at least in susceptible individuals[10].

Zookeepers have expertise as to what sort of conditions one ought to provide various animal species within the restriction of confinement in a zoological garden. As a rule of thumb, the ideal is to approach the EEA as closely as possible; in other words, to supply the type of conditions under which the species evolved. In the case of humans, optimal conditions are difficult to define, due to a superior capacity to adjust. Happiness can be achieved under a broad spectrum of life situations. Yet, for the average individual, certain aspects of the environment tend to be less constructive to quality of life. To the extent that these aspects reflect mismatches, they may be referred to as discords. Finding putative discords, and assessing them by further research, is a constructive strategy for improving society. One way to suggest candidate discords is to compare present living with current assumptions about the environment humans are adapted to live in.

The human brain appears to be particularly vulnerable to discords – due to its complexity, the fact that it requires substantial maturation after birth, and that the maturation takes place in response to environmental stimuli. This vulnerability helps to explain why mental disorders are considered one of the main health problems of Western societies[16].

It is difficult to retain happiness with a brain "scarred" by discords. In order to experience the default state of contentment fully, and presumably also to appreciate brain rewards, a well-adjusted brain is required.

The present text considers how the evolutionary model for quality of life can be applied in understanding the role of social life. The first objective is to outline what is known about the social nature of humans; i.e., the evolutionary forces that have shaped it and its impact on Darwinian Happiness. The second objective is to consider the concept of discords in relation to social life and explore to what extent this concept may help us to define strategies for improving quality of life. The third objective is an attempt to evaluate various alternatives to the present way of living. The final objective is to consider whether the evolutionary perspective can be used as a basis for advice on how society ought to be organized.

### THE SOCIAL NATURE OF HUMANS

#### Evolution of Bonding

Most experts agree that hominids evolved to live in a tribal setting. The tribe would be comprised of a number of adults of each sex, including several family groups, presumably totaling some 30–70 individuals. In most cases, individuals grew up together; however, some would come in from neighboring tribes, as exemplified by the movement of spouses. The individuals spent a great deal of time together and relied on each other for survival. Consequently, the tribe formed a strong social network. In fact, humans probably evolved some of the strongest innate tendencies to social affiliations of any mammal, in competition with species of canines, cetaceans, primates, and rodents. Interestingly, only select species within these orders are social, indicating that sociability evolved independently on several occasions in the mammalian lineage. Many species have adult females living together in harem groups, but it is rare to have adult males cooperating. Moreover, humans are unique in combining a highly organized social life with strong pair-bonding.

Research suggests that humans are endowed with the capacity to retain relations with 150–200 individuals[17,18]. Historically, the number presumably included one's own tribe plus members of neighboring tribes. Neighboring tribes would meet occasionally, as they depended on each other for exchange of mates, and probably exchanged information and tools as well. Only rarely would there be total strangers present, suggesting that in the absence of specific conflicts, an individual could trust the people with whom he interacted.

Although several species of monkeys are social, most apes do not form large groups[19]. The orangutans tend to be solitary; affiliation with the opposite sex is typically of limited duration and intensity. Gibbons and siamangs form monogamous relationships, while gorillas form family units that may include more than one mature female, but normally just one mature male. Only the two species of chimpanzees, the normal chimpanzee and the bonobo, form social groups akin to what we see in humans. As both the gorillas and the chimpanzees split with the human lineage some 5–7 million years ago[20], the gorillas slightly before the chimpanzees, it is reasonable to assume that social life in our lineage evolved over the last 6 million years. This implies that social behavior is a relatively recent addition to the human ethogram.

### Neurobiology of Social Life

The neurobiology and neurogenetics of mammalian social life has recently been reviewed[21,22]. Briefly, it is assumed that the underlying brain structures evolved early in mammalian evolution for the purpose of bonding between mother and child[11]. The structures were later co-opted for pair-bonding[23]. Additional bonding, such as between fathers and infants, and between adults of the same sex, most likely reflects an extension of the neurology used in these earlier forms of bonding. Thus, even though social life evolved independently in different lineages, the underlying brain modules appear to be related. In other words, once the neurology required for mother-child attachment was in place, expansion towards further social bonds could evolve relatively easily.

While all mammals have retained the bond between mother and child, only relatively few species form couples in the manner of lasting bonds between the parents. Several species form groups, but it is not obvious to what extent strong bonds between adults are required for animals to gather. In the herds of typical herbivores, the groups more likely reflect a herding instinct, rather than the actual recognition of other individuals as someone to cooperate with. However, in the social species, it seems highly likely that cooperation and concomitant emotional bonds are important for the social structure.

A large number of genes are presumably involved in orchestrating social behavior, yet it is striking that certain genetic diseases have a distinct impact on gregariousness. People with autism are withdrawn and unable to associate well with others, while those with William's syndrome tend to be hypersocial[24]. The former disease has a considerable genetic component, but the affected genes are unknown, while William's syndrome is caused by a more defined deletion, pointing to certain genes, such as GTF2I, as being particularly relevant for social behavior[25].

There has been a lot of focus on the role of the neuropeptides oxytocin and vasopressin in modulating social behavior[22]. The evolutionary root of these peptides can be traced to invertebrate species, where their role is related to procreation. In mammals, the peptides are implicated in various aspects of trust and bonding, including pair-bonding, but also in cooperation and sociability. Variations in the genes that code for these neuropeptides and their receptors have been linked to diseases that affect social behavior.

Bonding has also been associated with epigenetic changes. The best-known example is the mothering style in rodents, but the presence of peers during infancy can also have an epigenetic impact that may change the path of social development[26]. As to the former, if female rats or mice lick and groom their pups, their offspring will become less prone to stress and are more responsive to their own pups. This behavioral trait is passed on along the generations by epigenetic changes in the form of patterns of methylation that affect the activity of genes. It is conceivable that the human capacity for gregariousness can be influenced by similar changes and thus be inherited from parents to offspring in a Lamarckian fashion. In other words, the way we handle infants can have an impact for generations to come due to epigenetic changes. It is presumably more difficult to alter a course of events rooted in epigenetic changes, compared to changes that are purely neurological.

### Brain Rewards and Punishment in Social Life

The neural systems responsible for rewards in the form of pleasure, as well as punishment in the form of pain, are known to some detail[11,12,27,28]. The brain's reward circuitry includes neural structures in the ventral tegmental area using the neurotransmitter dopamine. Moreover, the nucleus accumbens plays a critical role in pleasurable states. The ventromedical prefrontal cortex and amygdala are also major dopaminergic targets that have been implicated in reward processes. The original function of these systems was presumably to encourage behavior such as eating and sexual activity. The pain network consists primarily of the dorsal anterior cingulate cortex, insula, and somatosensory cortex, with subcortical contributions from the periaqueductal grey and thalamus.

For the present purpose, it is of particular interest to note that the ups and downs associated with the emotional response to sociopsychological events rely on much the same neural circuitry that underlies the typical pain and pleasures caused by physical stimuli[27,29]. For example, experiencing envy of another person's success activates pain-related circuitry, whereas experiencing delight at someone else's misfortune activates reward-related neural circuits. Similarly, feeling excluded or being treated unfairly activates pain-related neural regions[30,31]. On the other hand, positive social feelings, such as getting a good reputation and being cooperative, offers rewards similar to those one gets from desirable food[32,33]. It is worth noticing that the act of giving, as in charitable donations, can cause a stronger activation of the reward network than receiving the same sum of money[34]. In fact, the rewards of giving and acting on empathy can improve personal well-being[35,36].

Both acts of aggression and acts of compassion can be highly useful for survival and procreation and, as such, would be expected to elicit brain rewards. As discussed in more detail elsewhere[37], compassion, somewhat surprisingly, appears to be considerably more rewarding than acts of violence. This disparity may be related to the two types of behavior being shaped at different periods of our evolutionary history. At earlier stages of mammalian evolution, the behavior repertoire was presumably instigated more by instinctive tendencies than by rewarding sensations. As consciousness and the concomitant capacity for free will expanded, incentives in the form of rewards became more important, simply because the free will might otherwise tend to "overrule" the behavioral tendencies laid down to benefit the genes. Human social propensities most likely evolved much later than our aggressive instincts, offering a possible explanation for why hugging people seems to be more useful for the purpose of feeling good than hitting people. Obviously, other factors, such as cultural influence, also impact on the feelings instigated by social or antisocial behavior.

The main point is that social relations offer a rich source of brain rewards due to the importance of associating with others. The rewards include those we sense when falling in love, feeling love, being with friends, and engaging in the fate of others with compassion. Relations are arguably our most potent source of good feelings.

### DISCORDS ASSOCIATED WITH SOCIAL LIFE

#### An Achilles Heel?

The tribal way of living began to disappear 10,000 years ago with the advent of agriculture. Social relations within the community have changed dramatically since then. The lack of a tribal setting may, in fact, be the most significant discord between present life and that of the human EEA. Without our strong, innate social propensities, large-scale societies would never exist. And yet, due to the difficulties of avoiding social discords, human interactions may prove to be an Achilles heel for the human species.

In modern society, there are numerous mismatches associated with how we evolved to interact with other individuals. For example, the close-knit tribal world of the Paleolithic era has been replaced with nuclear families, and a relatively weak and unstable social network. In the tribal world, there was always someone around for comfort, and relations typically lasted for life. The culture, including morals and ways of behavior, was stable, implying that people knew how to deal with each other as well as what to expect. Transactions were among affiliates, so the individual could trust that social contracts were heeded. Today, most people experience daily encounters with strangers and we are forced to deal with a range of people with whom we do not have personal relationships and who we may never meet again.

Some of the mismatches may not matter, or actually offer advantages, while others prove to be discords. It is, however, difficult to distinguish between mismatches and discords. Some aspects of modern living may be sensed as an advantage, but still have a negative long-term affect on the psyche. A possible example is the feeling of "freedom" associated with not having any close ties or commitments. Moreover, a discord may affect only a subset of the population. As discussed below, the concept of social discords may help to explain several quandaries of modern society, including stress, loneliness, lack of belonging, the high prevalence of diseases such as anxiety and depression, and the numerous conflicts between individuals. A suboptimal quality of life is the likely consequence of these problems.

### Possible Impact of Discord Community Relations

According to recent estimates, 31–50% of the population of industrialized nations suffers from a mental disorder at some point in life, whereas 17–33% have had a diagnosable condition during the last 12 months; in fact, neuropsychiatric conditions account for a quarter of all disability-adjusted life years[38,39]. Diagnosable mental diseases are presumably only the tip of the iceberg as to mental agony and suboptimal quality of life[10].

It seems unlikely that the above data reflect the default state of the human mind, as many of the mental conditions would be expected to be under considerable negative selection in a tribal, hunter-gatherer setting. A reasonable interpretation is that the high prevalence is due to the combination of two factors: brain modules that are sensitive to discords and the presence of discords affecting these modules. Some individuals are genetically more prone than others to develop mental diseases, but the environment is probably responsible for most of the problems[14,40].

Depression and anxiety-related disorders tend to top the lists of mental diseases. Anxiety is related to inappropriate triggering of the fear module, while depression is associated with excessive activity of a low mood module[40]. Some of the other mental problems may be secondary consequences of disturbances in the fear and mood functions. Certain sleep problems are, for example, caused by anxiety, while misuse of drugs and alcohol may be related to a low mood. Therefore, it seems likely that mood and fear represent brain modules that are particularly vulnerable to discords. The responsible discords would be expected to involve social interactions and to constitute a considerable burden to the present population.

In order to comprehend the likely effect of discords, it is relevant to be aware of how the functions of a human body develop. Both muscular and mental modules tend to increase in power and performance upon stimulation and use, a fact well known to those involved in body building. The fear and low mood modules are presumably no exception, as is well demonstrated in the case of fear[11]. Within the brain, exercising a module will tend to imply that the module performs better and/or has a stronger impact on consciousness. However, while improving the size of muscles or becoming a better chess player may be desirable, an abnormal dominance of the fear and low mood modules is not. Discords causing frequent or unbalanced stimulation of these modules are a likely cause of unwarranted activity and ensuing mental disorders.

It has been documented that stressful conditions for infants, such as abuse or separation from the mother, can lead to anxiety- and mood-related disorders in both humans and animals[41,42]. However, the prevalence of these disorders in humans appears to be much higher than the prevalence of serious child abuse or neglect. Moreover, most patients do not report such a background. Thus, even in the case of what is presently considered a normal upbringing, there are likely to be practices and cultural traditions that contribute to a decline in mental health.

The role of early childhood discords in explaining the high prevalence of anxiety has been discussed in detail elsewhere[43]. Briefly, Stone Age people presumably would stay in close proximity to their children, carrying them around during the daytime and sleeping next to them at night, as tribal people tend to do today[44]. In modern societies, infants typically spend much time without a sensation of where the parents are, as exemplified by the sleeping arrangements where the infants are placed in their own cribs and often in separate rooms. If children cry when put to bed, a dominant line of thought has been that it is best to ignore their crying in order to teach them to sleep alone[45]. Following this advice, the baby will eventually stop crying; however, the situation may, over time, spur excessive development of the fear function. Activation of fear, particularly of the type referred to as separation distress, may also follow as a consequence of other aspects of modern living, such as the use of daycare centers.

It is important to note that the question is not whether the environments the babies are offered are hazardous. Both daycare centers and modern housing may very well be safer than Paleolithic camp sites. The point is that children are prone to respond to the absence of parental proximity as a threat, while not responding to more imminent dangers simply because they have evolved to rely on the parental effort to escape.

Another relevant mismatch concerns the amount of skin-to-skin contact that infants receive. In the Paleolithic age, there would be limited use of clothing, and more handling and carrying against the body[46]. Skin-to-skin contact is known to calm people[11,47]. Thus, a decrease in the dose of either nursing or other forms of touch may contribute to anxiety disorders.

Frequent encounters with dominant individuals, such as teachers, bosses, and strangers taking a dominant position, may lead to an abnormal expansion of the low mood module, which can contribute to the development of depression. The lack of a proper social network most likely aggravates the problem.

Discords are also likely to amplify conflicts, both at the group level and at the individual level. The "tragedy of the commons" is the story of how shared pastures get destroyed as a result of overgrazing because farmers are unable to limit the number of animals. The story is used as a metaphor for a general lack of collaboration[48]. In the Stone Age, collaboration was presumably easier due to the closer ties between the individuals involved. Conflicts did arise, particularly in places with high population density, but on the whole, the further increase in population and concomitant decrease in closeness within communities are likely discords affecting the level of collaboration achieved. As to the individual level, while conflicts in the Stone Age probably tended to find resolution, today they can last a lifetime. You do not depend on your neighbor for survival, neither do you have a life-long commitment with him; consequently, the quarrels that arise are more difficult to settle. Unresolved disputes can have a considerable negative impact on life.

Possibly the most obvious discord, as well as the most common complaint, is simply one of loneliness and lack of belonging. People do not develop positive community relations of the strength and duration evolution has shaped our mind to expect.

The Internet and cellular phones have made communication easy. Nevertheless, the technology also implies a likely discord. Talking face-to-face, the physical presence, the smell, and the touch – these are the social settings our minds are primed for and electronics cannot replace them completely. The modern tools of interaction can be useful, but if they end up reducing physical proximity and contact, they may promote mental disturbances.

### Cities – Just Another Narcotic?

If we were able to outline the various discords associated with modern civilization, could we expect people to take the necessary action to improve their lives?

Examining the expansion of cities is relevant for answering this question. Urban areas would be expected to be less in line with the human EEA compared to rural living, yet people flock to the towns. This is not just a recent trend. Catalhöyük, in Turkey, was established some 9,000 years ago and had 10,000 inhabitants at the most[49]. The houses were so densely packed that entrances had to be through the roofs. Apparently, the inhabitants did not have division of labor and lived as hunter-gatherers with only rudimentary agriculture. In other words, people moved together even though their subsistence suggested that a dispersed habitation would ease the gathering of food, and even in the absence of the advantages associated with the creation of specific vocations.

It is possible that the Catalhöyük people gathered for safety. The area presumably started to feel population pressure and it is easy to imagine swarming hordes of bandits attacking small tribal units. Alternatively, the city could have been based on a religious movement, but archeologists have not found obvious signs of temples or other religious structures. There are many reasons why people move to cities, yet it is tempting to speculate that one of them is that crowded spots work as a "superstimulus", catering to our social nature, akin to how a bird prefers the bigger plastic egg rather than the real egg. We have an instinctive tendency to seek places where there are many people.

To the extent that the above interpretation is correct, it supports the argument that humans, or for that matter other species of animals, do not necessarily choose what is best for long-term quality of life. If the environment is different from the EEA, behavior is likely not to even favor what is best for survival, as in the example of the plastic egg. The present human environment is considerably different from the human

EEA; consequently, we are not always expected to make the best choices. In fact, this notion may help to explain why so many people have physical or mental health problems, spend their lives quarrelling, commit suicide, or become drug addicts. We are influenced by instinctive tendencies that are not tuned for the present way of living.

A related phenomenon has been described for "rural" and "urban" rhesus monkeys in India[50]. The rural monkeys live in troops that range over the forested countryside, eat a variety of fruits and vegetables, and rarely fight with each other. Their urban cousins gather in temples where the Hindus feed them. Although the food in the temples is typically more abundant than in the forest, the urban gangs fight and quarrel a lot. The rural monkeys seem to live a better life, yet there is no indication that the temple gangs wish to move back to the forest. Temple life most likely involves several discords, with concomitant stress: it is more crowded, the monkeys lack a regular troop to belong to, and the food in the temple tends to be concentrated in particular locations, rather than being more evenly dispersed, thus causing more fights. The point is that the monkeys choose to live there, even though it does not boost their quality of life. Humans are not necessarily that much better at making intelligent decisions; immediate satisfaction is closer to our heart than optimal choices[51].

One may also ask, if loneliness is a common problem, why do so many emphasize their need for personal freedom and independence? Possibly, the strong social impact experienced in a typical crowded society causes a reaction towards seclusion, particularly because the majority of signals received are likely to be either unfriendly or neutral, rather than friendly. Furthermore, the situation of having no one to take responsibility for, or share responsibility with, has obvious appeal. The fact that the ensuing freedom tends to come at a cost, in terms of potential loneliness and a lack of people to turn to when in need, does not prevent the desire for independence. Freedom seems to reflect more of an immediate desire, while geniality is more of a long-term investment.

Apparently, humans are not inclined to perform evaluations of the sort required for optimizing happiness. Some decisions follow from "button-pushing situations", such as hitting back when somebody takes a punch at you. However, even more deliberate actions are not necessarily beneficial either as to long-term advantages or immediate rewards. The latter point is accentuated in the case of drug addicts. Their cravings continue even when the addict no longer experiences any pleasure upon taking the drug. In order to explain this observation, Robinson and Berridge[52] introduced *wanting* and *liking* as two brain modules related to motivation. The function of the wanting is to wet the appetite and to energize the mind for tasks ahead, while the liking constitutes the main part of the rewards. The wanting is highly active in the cravings of an addict, but although it may be linked to positive sensations, this is not necessarily the case. Even when unhooked from rewards, the wanting can still exert a strong influence on decision making, as reflected in the problem of getting rid of an addiction. The lure of the cities and the demand for personal independence may reflect activation of the wanting module.

An attempt to restrict people's freedom of choice for the purpose of improving their quality of life is unlikely to succeed. The intention should rather be to enlighten people as to what is inherent in human nature and what options are likely to offer the best long-term benefits. Humans do have the capacity to let intelligent, conscious decisions overrule innate tendencies.

### ALTERNATIVES

#### Are There Alternatives?

The above discussion suggests that Western civilization has not found the optimal solutions for organizing a community. The obvious question, then, is whether there are realistic alternatives. In order to explore this issue, I shall take a look at some options that might point towards possible improvements.

One obvious approach for finding better ways of handling human resources is to try to understand how Paleolithic tribes lived. Describing the human EEA, i.e., the way of life we are adapted to, might help us to avoid some discords. The few remaining tribal people presumably live under circumstances closer to the EEA compared with people in industrialized nations. A first question is whether these people are happier and, if so, does their tribal way of living suggest alternative strategies applicable to industrialized society?

Many people feel that something is wrong, a dissatisfaction that has led to the formation of a variety of alternatives based on personal initiatives. These alternatives – typically referred to as either intentional communities, communes, or ecovillages – offer a second approach. The question is not necessarily whether they have found a complete solution that can be transferred to large-scale societies, but whether there is anything to be learned, any cultural elements to be exploited.

The third approach is to look at variations in present large-scale societies. There are considerable differences as to how nations function, reflected, for example, in the indexes of national happiness[7,53,54]. In fact, the variation observed concerns many aspects of social life, including parameters such as generosity, nastiness, and altruistic punishment[55,56]. A variety of conditions contribute to these differences, some may point towards preferred ways of organizing a nation.

The three types of options presented above – tribal people, intentional communities, and features of particular countries – are all of interest for the topic of the present text and will therefore be discussed in the following sections.

### Tribal People

Although the way of life of present tribal populations may have less discord than industrialized societies, their situation is not equivalent to that of the Paleolithic hunter-gatherers. Still, comparing the quality of life in tribes with that of surrounding populations should be of considerable interest. Unfortunately, to the author's knowledge, nobody has made any serious attempt at performing such a comparison.

The lack of data is partly due to technical problems. It is difficult to compare happiness between highly divergent cultures. The questionnaires used to assess subjective well-being are not easily translated in a way that makes the answers equivalent. Furthermore, health statistics for tribal groups are rare due to the limited health services. Consequently, although the anthropological literature is rich in descriptive work on tribal people, it is short of efforts assessing their quality of life.

The author has personal experience from visiting some tribal groups: Maasai and Hadza in Kenya and Tanzania, as well as aboriginals in Malaysia, Philippines, and Australia. The two African groups have a strong awareness of their own situation and culture. Moreover, they are actively engaged in retaining the main features of their traditional life, albeit with some provision for the advantages of greater society in the form of tools, health care, and, at least for the Maasai, education. The impression was that these people were at least as content as the surrounding populations.

In the case of aboriginals, however, there seemed to be less of a drive towards maintaining traditional ways; in fact, the majority had apparently moved to mainstream society where they typically ended up far down on the social ladder. With the possible exception of Australia, the tribal living appeared to be sustained less by conscious effort than by limited options. Healthwise, the aboriginals of Northern Australia, for which there are some data, appear to be worse off than the residual population[57]. Overall, the impression was that while the African tribes probably had superior quality of life, the opposite may be the case for the aboriginals. These impressions are, of course, based on subjective interpretations and a limited amount of observations.

Even if relevant data did exist, it would be difficult to deduce much as to advantages of living a life closer to the EEA. One problem is that all the remaining tribal groups are, to a greater or lesser extent, influenced by the modern way of life. They tend to be aware of material advantages and possibilities of the outside world, and they are usually displaced into marginal areas, making their traditional way of life difficult to sustain. In other words, they experience a range of nuisances that the ancient hunter-gatherers did not have to deal with. On the other hand, they may take advantage of some of the tools and health care of the modern world. The point being that assessing the quality of life of present tribal people has only limited value as to understanding the potential for happiness associated with ancient tribal life.

It is not feasible to implement the human EEA, with small tribes and the sparse settlements required for sustenance, on a global scale with the present world population. Neither would tribal life make it possible to retain the technical and medical advantages of modern civilization. Yet, to some extent, it may be possible to utilize elements of tribal life in large-scale societies. For example, one might try to establish conditions conducive to the generation of smaller and closer-knit social groups.

### Intentional Communities

Over the last century, thousands of initiatives all over the world aimed to create alternative ways of living. Most were small in scale, from a few family units to a couple of thousand inhabitants. Some initiatives proved to be short-lived, but others have survived for decades and prospered in terms of life satisfaction. A number of them can be found in the Online Communities Directory or the Global Ecovillage Network.

Although these initiatives have led to a variety of ways of living, certain features are common[58]. These features include a down-scaling of the community, so that the individual may form emotional bonds with the other members, and having social life revolving around the communal group, rather than just the nuclear family. As such, the intentional communities incorporate attributes that resemble the tribal way of living. Other typical features that probably approach tribal conditions include limited hierarchy, decision making by consensus, and shared work leading to a complete or partly pooled economy.

Most initiatives have been based on spiritual or ecological ideas, i.e., they grew out of a desire either to live with people of similar religious devotion or to create a more sustainable way of life. More or less all, however, have an expressed purpose to obtain a better social setting with deeper and more satisfying relationships. Kirby's[59] description of the ecovillage at Ithaca offers a typical example.

Comparing health and well-being between communes and surrounding populations should be a lot easier than in the case of tribal people, but relatively few studies have been performed. The kibbutzim of Israel have been examined and the reports suggest that health, well-being, and life expectancy is improved among members compared to the outside population, at least when comparing the elderly[60,61]. Social life is assumed to be an important contributor to this effect.

The Hutterite population residing in Canada is another interesting case. They have built their religious societies on the notion that groups of up to 100–150 people can form well-functioning units with personal ties among all members[62]. They recognize that social pressure can be used to control behavior and resolve conflicts within units of this size, which is in line with the previously discussed research on the human capacity to form relations. A report found significantly fewer psychoses among the Hutterites compared to the surrounding population[63].

Although there are very few formal studies that explore advantages and disadvantages as to quality of life, there is considerable literature of a more anecdotal type, which suggests that life is better in intentional communities. After visiting several, rather different communes, my personal experience points in the same direction. Although there seems to be a tendency for the alternative options to recruit individuals who have difficulties adjusting to large-scale society, and who may not be the easiest persons to care for, the impression is that most communities are reasonably good at solving problems and creating an atmosphere where people thrive. The overall impression is that many people appreciate the lifestyle offered. Besides the possibility to live according to religious or ecological philosophies, the main advantage seems to be the interpersonal relationships and social networks that communal living caters to and provides.

Many of the more successful communes appear to be based on religious or spiritual doctrines. Religions not only have a potential for making people happy[64,65], they are also a potent means for creating common rules that people accept. In fact, evolution possibly shaped the human brain in the direction of religiousness for the purpose of improving social cohesion[66].

### Unusual Nations

There are comprehensive reports that evaluate the happiness, or subjective well-being, of different nations[7,53,54]. The overall inferences based on these comparisons are that social connection is a prime factor; spirituality helps, as does a well-organized nation with ample health care and advanced technology. Other positive factors include a sense of being in charge of one's own life (exemplified by democratic institutions), commitment and engagement in society (whether being in the direction of politics, religion, or sport), and compassion (revealed, for example, as membership in charitable organizations). An interesting feature is that island nations tend to come out better than those on the continents. The latter point may reflect that islands typically have smaller and more compact communities and, being more isolated, people probably also feel closer.

Most of the features listed above are in some way related to community relations. Two case stories will be presented, that of Japan and Bhutan, in order to look further into the role of social life.

Japan was brought out of its self-imposed isolation late in the 19th century. The country never had much in the way of natural resources and at the time was industrially far behind the Western world, yet Japan managed to perform exceedingly well in economic terms. Prosperity does not necessarily improve happiness, but it is noteworthy that the country succeeded in a task most countries strive very hard to achieve, albeit with less success. Understanding Japan's accomplishment is of interest as it may help other countries to reach whatever aims they give priority to; for example, improving the quality of life for their citizens.

Traditional Japanese society has features related to the tribal way of life[67]. Factories and offices in Japan are organized as units with close-knit ties. Consequently, the employees feel a moral responsibility for the welfare of each other and the work place. Moreover, the whole country can be seen as a "supertribe", in that the Japanese appear to have a particularly strong sense of belonging to their nation. Behavior is governed more by morality, or honor, than by law, as illustrated by the observation that the U.S. has five times as many lawyers per capita. It seems likely that this way of organizing the country was vital to its accomplishment. Presently, Japan scores above average on happiness scales, but below other countries with equal wealth such as Western Europe[7]; however, the traditional features of Japanese society seem to be disappearing.

Another interesting country is that of Bhutan. This nation is today in a position akin to what Japan was at the end of the 19th century. Bhutan is economically and industrially underdeveloped, has limited natural resources, and is about to come out of a self-imposed isolation. There is, however, at least one important difference. While the global sentiment of the 20th century was towards economic development, the present trend seems to lean more towards rating happiness as a primary objective. Japan focused on economic success, but Bhutan has actually taken a leading stance in the direction of an alternative priority. The king has announced that in his country, the focus should be on gross national happiness rather than gross national product. The country now appears to be among the top developing countries on the happiness scale[53].

The *Centre for Bhutan Studies*, which was started for the purpose of setting priorities and devising strategies for reaching them, suggests nine relevant domains (according to their website): psychological well-being, time use, community vitality, culture, health, education, environmental diversity, living standard, and governance. The "community vitality" domain focuses on the strengths and weaknesses of relationships and interactions within communities. It examines the nature of trust and belonging, the vitality of caring relationships, security in the community, and the propensity towards generosity and compassion. The important point is that it is possible for a nation to focus on happiness and that such a focus may actually improve the well-being of the citizens. Moreover, well-functioning communal units, with strong social ties, appear to be particularly relevant for improving quality of life.

## DISCUSSION – THE EVOLUTIONARY PERSPECTIVE

### Obtaining Rewards – Avoiding Discords

The evolutionary perspective on happiness suggests two types of brain modules to be of particular importance: that of rewards and punishment, and that of default contentment. Social relations are among the most potent triggers of rewards, but can also generate punishment, for example, in the form of depression, social anxiety, and rejection. Moreover, present social life may constitute one of the most problematic forms of discord and, consequently, may be responsible for a suboptimal quality of life. Social discords are expected to disrupt the default contentment, increase the chance of stress and mental disorders (particularly those related to anxiety and depression), and make it more difficult to appreciate the rewards associated with social life (see Table 1). Thus, as a rule of thumb, society ought to encourage a community setting that approaches the circumstances under which humans evolved.

There are, however, reasons to break this rule of thumb occasionally. For one, not all mismatches are discords. Moreover, even the mismatches that contain an element of discord can offer benefits. In order to sustain the advantages of a modern, industrialized society, some compromises are required, implying that discords should be tolerated if they come out positive in a cost-benefit analysis. In fact, by using the opportunities available in modern societies, it may be possible to offer a better communal life than what was the case in the tribal setting. For example, as will be discussed in the next section, it should be possible to induce compassion and reduce aggression. Understanding the innate tendencies associated with social life, as well as the modules of the brain that impact on happiness, is relevant to this endeavor.

The objective should not be to recreate the tribal setting in an industrialized society, but to approximate those features of tribal social interactions that are likely to improve well-being. Intentional communities do so on a small scale. The traditional Japanese culture suggests that it is possible to benefit from employing tribal units at the work place in an industrialized society. Establishing strong social units, either based on living quarters or work, is expected to improve both productivity and quality of life. In fact, improved well-being is expected to boost productivity regardless of why people are happier; Bhutan surprised the world by being second in the world as to economic growth in 2007[53].

Measures taken need not be radical. Improvement is possible by minor changes; for example, making it easier for people who work together to learn to know each other through informal interactions. Anything that activates the positive side of the social affect system should promote bonding, and thereby promote cordiality and happiness. The clue is to be able to handle conflicts that arise and to promote a genial atmosphere. Grooming in social primates is well known to stimulate positive relationships and has been shown to boost opiate-related reward mechanisms in the brain[68]. It has been suggested that language evolved as a form of "grooming" in that it helped to facilitate communal relations in large groups[69]. While physical grooming typically involves only two individuals at the time, oral grooming can engage several persons simultaneously. Small-talk at work may have more value than what is often recognized.

Social life needs to be learned. Children that grow up in the absence of, or with minimal, social contact tend to be mute, bestial, and egocentric[70]. To learn to socialize is somewhat like learning to speak. We have an innate propensity, a template, but in order to develop our social capacity, we need suitable input. Interacting with others molds our social competence. Play and conflict resolutions are important forms of interaction. If the social conditions for infants are discord, the situation is unlikely to yield an adult with optimal social performance. In other words, society should be particularly mindful as to how children are raised.

In Paleolithic tribes, there was no difference between paid work and work at home; everything was done with, and for, those with whom one had affiliations. Work was presumably not even considered to be "work", but just a part of life. Thus, family concerns, community affairs, and the various activities were continuous elements in the daily round of life. The products of labor were either consumed by the producer or shared/exchanged with associates. It tends to be satisfying to work for your personal needs and for the needs of those who are close to you.

Many communes try to approach this situation. Unselfish sharing of labor and resources is, however, difficult to implement in an industrialized nation. The communist countries tried, but did not succeed very well. Apparently, strong community relations need to be established first. Still, a nation may make the conditions suitable for local communities to form and flourish based on these principles.

### The Pendulum Analogy

It is within human nature to be revengeful and sadistic, and consequently, to devastate the quality of life of others – and typically personal well-being as well – by hostile behavior. For the purpose of maximizing the quality of life of the population, it seems obvious that violence ought to be discouraged, while compassion encouraged, even beyond what one may argue is closer to the innate characteristics of human nature. Thus, although relevant measures may be construed as mismatches, they should still be encouraged.

Human behavior can be modulated, but our versatility is restricted by a set of "elastic limits" defined by the genes. A pendulum may be used as an analogy to illustrate the relationship between innate dispositions and the actual observed level of benevolent behavior (Fig. 1). In this analogy, moving the pendulum to the right reflects an increase in benevolence, while movement to the left implies more aggression. The actual position of the pendulum depends on two factors. The first is gravity, which is analogous to the pull of the genes. The direction of this "field of gravity", however, depends on the conditions under which we live. In a natural environment, the gravity pulls straight down. In this case, the default levels of respectively aggressive and cooperative behavior mirror the balance evolution has brought to our genes. Thus, the natural position implies a fair share of selfish and nasty behavior. On the other hand, when living in discord conditions, the field of gravity is tilted to the left, i.e., toward aggression, due to the associated stress and unbalance created. The pendulum will, of course, tend to follow the pull of gravity. The second factor is our capacity to grab the pendulum and drag it in the desired direction, which corresponds to how we manage human resources. In other words, in order to move the pendulum towards benevolence, we have two options: either we can change our way of living towards what our genes are adapted to, and thus tilt the field of gravity, or we can try to haul the pendulum away from its point of equilibrium towards compassion. Both options are possible and should be pursued.

There are a number of ways in which society can stimulate our caring and cooperative tendencies. Schools and the mass media offer excellent opportunities to nourish the public with stimuli designed to foster compassion. Simple reminders of being kind and smiling at others can have an impact. Unfortunately, it is a lot more difficult to avoid stimuli that provoke our aggressive tendencies. What a government can do is to create the best soil for culturing positive stimuli. The most cooperative societies also appear to be the most generous; thus, collaboration apparently fosters geniality[55].

In theory, it should be possible to induce more kindness than human nature suggests and thus create a society with more compassion than what was typical for Stone Age tribes, but the task requires a resolute effort. Holding the pendulum away from the center of gravity demands a constant input of "energy" in the form of initiatives designed to promote sociability.

Handling the various innate tendencies associated with social life, with the overall purpose of improving people's quality of life, is one of the most important challenges of present societies. One may wish humans were different, that our true nature was all kindness, but it is not.

### Conclusion

The ancient Greek philosopher Epicurus promised his followers happiness if they would live with him in a commune called The Garden. There they would be surrounded by friends and have a tranquil, self-sufficient life in the absence of anguish and fear. Can the evolutionary perspective help industrialized nations to fulfill a similar ambition?

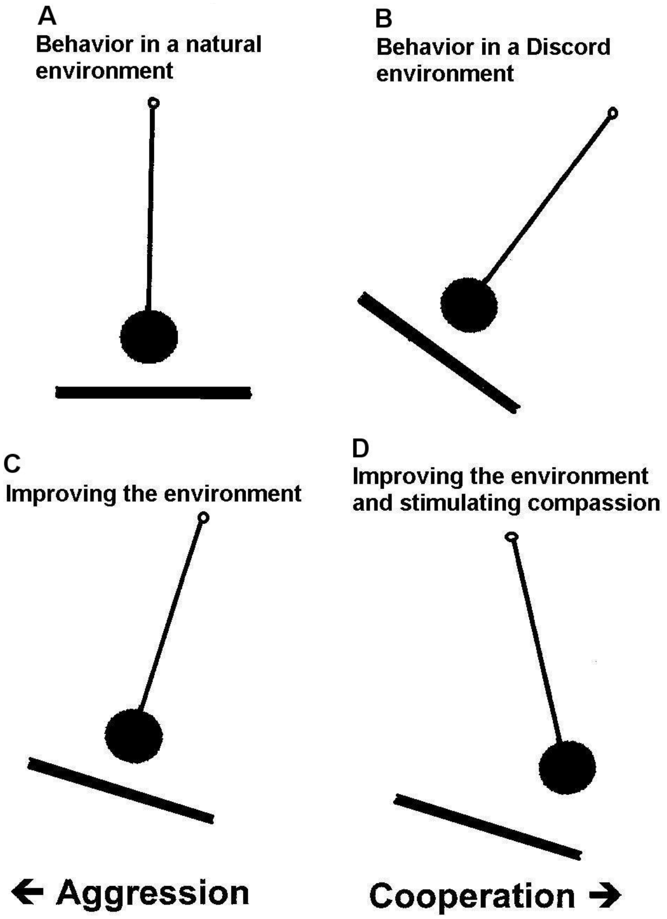

**FIGURE 1**. The pendulum as an analogy to aggressive vs. cooperative behavior. In the natural environment, the pendulum will follow the pull of gravity (A). More aggressive behavior is a likely consequence of a stressful, discord environment (B). It is possible to improve cooperation either by creating an environment with less discord (C) or by actively pulling the pendulum towards geniality (D).

Homo sapiens are genetically a homogenous species, due to having a shared common ancestor dating back only some 100,000 years; moreover, most of the genetic variation is found *within* each of the different subpopulations[71,72]. Innate tendencies can, therefore, at least for practical purposes, be considered the same for any present, past, or future population. That, however, does not imply that the biological perspective points towards a single, optimal solution for all inhabitants of the world. For one, cultural traditions impact on the human phenotype and should be taken into account, and two, individual differences exist, implying that the optimal for a nation may be to have a certain variety of ways of living to choose among. Moreover, we do not have complete understanding of what the human EEA is like, or how evolution has shaped the human mind; thus, advice based on current models may be flawed. Yet, our understanding of human nature is sufficient to indicate some guiding principles.

Intentional communities can be seen as a modern way of approaching tribal life, albeit these initiatives rarely, if ever, grow out of an awareness of the evolutionary perspective. The rationale for choosing solutions in line with biological thinking is typically either intuitive or by trial and error. The concord between the practical approach of the communes and the more theoretical solutions suggested by biology can be taken as support for the relevance of both.

Most communes put a lot of energy into developing communal relations. They consequently offer interesting insight both as to the importance of the social network and as to ways of achieving positive relations. There are many features that can help to make a group of people function well together. To a large extent, it is a question of details, as in the following examples:

- Simply the way of saying hello to each other can make a difference. In the Damanhur community of Italy, inhabitants use "*con te*" – "for you" – as a greeting, to signify that they care about each other.
- More skin-to-skin contact is advisable, as exemplified by cultures where people hug each other.
- Sharing food and agreeable experiences will help to advance relationships.
- Decision making by consensus, rather than voting, generates more support for the initiatives in question.

The present variety of cultural elements that cater to social life may not cover all possible alternatives, but probably include the more useful options. Trying to evaluate these options scientifically should be of considerable interest. As in the case of Bhutan, the aim of governance can be to implement elements that together improve quality of life.

Bonds between people play a key role in human life. As has been documented[73], people readily form social attachments and resist the dissolution of existing bonds. Mental and physical health problems are more common among those who lack close personal relationships; i.e., without caring relations, people are more likely to be unhappy, depressed, and anxious[74]. Happy people tend to foster more compassion. Ideally, this should be a positive feedback cycle: When people are satisfied, it is easier to induce compassion and geniality, which again should help the individuals to improve their quality of life further. In support of this idea, it has been shown that happiness tends to spread in a community[75].

The point about socializing can, to some extent, be implemented on a national scale, as exemplified by traditional Japanese society. Solutions for whole nations are important because it seems unlikely that the majority will settle in communes. Moreover, the current trend may not even favor communal living; in Israel, people move from the once-successful kibbutzim to the independence of family life in the cities[76]. The biological perspective suggests that it is easier to create close ties and concomitantly improve quality of life in a small commune. It is, however, possible to argue that the advantages of living in a city carry more weight. Then again, as discussed above, people may tend to prefer cities and independence even though the sort of life chosen decreases their expected level of happiness.

Few communes are likely to survive without the support of mainstream society. Many of the products used, from health care to mobile phones, are based on the industrialized world. Thus, it may neither be desirable, nor feasible, to have everybody live in this sort of small-scale community.

Caring for the young is of particular importance. The first years of life coincide with the most active period of brain development. Discords affecting infants are consequently expected to be a bigger problem than discords affecting adults. As discussed in relation to anxiety, the evolutionary perspective has distinct recommendations as to how one ought to care for the young.

Religion may help by creating social rituals, making people adhere to moral rules, and offering people the chance of finding comfort in a god[65]. The usefulness of religion in this respect is likely to explain why religious communes have a better field record of survival compared to purely secular communes. Many monasteries have existed for centuries, if not millennia.

The New Economics Foundation proposes that the purpose should not just be happy citizens. Their *Happy Planet Index,* used to rank the quality of nations, is based on the following formula: *well-being* $\times$ *life years / ecological footprint*[53]. In other words, it is not just the immediate quality of life that matters, but also how many years you can enjoy it, as well as the possibility for future generations to obtain an equal quality of life.

Whether these additional qualifiers are taken into account or not, social life is a key factor. So is an understanding of the innate tendencies evolution has put into the human mind. Our great feats of engineering, from understanding genetics to sending a man to the moon, are the easy tasks. The real challenge in shaping the future world lies in dealing with human nature. The key resource is human capital. Present ways of living tend to include several discords associated with communal relations and social life. It should be possible to reduce these discords, and thereby improve mental health and quality of life. However, most of the recommendations suggested by the evolutionary perspective have yet to be confirmed by directed research. With more verified knowledge, the government can enlighten people as to the consequences of various choices, and stand a better chance at creating conditions favoring successful communities with happy citizens.
